# Impact of Intrauterine Growth Restriction and Placental Insufficiency on Nutritional Outcomes of Extremely Low Birth Weight Infants

**DOI:** 10.7759/cureus.31611

**Published:** 2022-11-17

**Authors:** Shreyas Arya, Amara Uzoma, Aimee Robinson, Alvaro G Moreira, Sunil K Jain

**Affiliations:** 1 Division of Neonatal-Perinatal Medicine, Department of Pediatrics, Wright State University Boonshoft School of Medicine, Dayton, USA; 2 Department of Obstetrics and Gynecology, University of Texas Medical Branch at Galveston, Galveston, USA; 3 Division of Pediatric Pulmonary Medicine, Department of Pediatrics, The University of Tennessee Health Science Center, Memphis, USA; 4 Division of Neonatal-Perinatal Medicine, Department of Pediatrics, University of Texas Health Science Center at San Antonio, San Antonio, USA; 5 Division of Neonatology, Department of Pediatrics, University of Texas Medical Branch at Galveston, Galveston, USA

**Keywords:** neonatal growth, conjugated hyperbilirubinemia, total parenteral nutrition, doppler ultrasonography, neonatal nutrition, elbw neonates, umbilical artery, aredv, placental insufficiency, intrauterine growth restriction

## Abstract

Introduction

The aim of our study was to assess the impact of intrauterine growth restriction (IUGR) and placental insufficiency (PI) on the nutritional outcomes of extremely low birth weight (ELBW) infants.

Methods

We conducted a six-year retrospective case-control study that included 117 ELBW infants. Of these, 58 infants had IUGR and 59 were born appropriate-for-gestational age (AGA). Infants with IUGR were further divided based on the presence or absence of PI, as determined by umbilical arterial doppler velocimetry on serial ultrasounds.

Results

IUGR infants with PI had the lowest enteral calorie intake at 28 days of life (DOL) (median intake- IUGR+PI: 32 vs IUGR-PI: 93 vs AGA: 110 kcal/kg/day; p-value 0.011) and at 36 weeks corrected gestational age (CGA) (median intake- IUGR+PI: 102 vs IUGR-PI: 125 vs AGA: 119 kcal/kg/day; p-value 0.012). These infants also trended towards requiring a longer duration of total parenteral nutrition (TPN) (median duration - IUGR+PI: 35 vs IUGR-PI: 25 vs AGA: 21 days; p-value 0.054) and higher incidence of conjugated hyperbilirubinemia (IUGR+PI: 43% IUGR-PI: 29% vs AGA: 16%; p-value 0.058), but these results did not reach statistical significance. Despite challenges with enteral nutrition, IUGR infants with PI showed good catch-up growth and had higher growth velocities over the first month of life, compared to AGA controls.

Conclusion

IUGR in the presence of PI is associated with significantly poorer enteral nutritional outcomes in ELBW infants. However, with the support of optimal parenteral nutrition these infants showed good catch-up growth.

## Introduction

Intrauterine growth restriction (IUGR), in broad terms, is a condition in which fetal growth is less than its genetic potential. This growth potential depends on a variety of factors, including gender and race [1]. In clinical practice, the term small-for-gestational age (SGA), defined as birth weight < 10th percentile for gestational age (GA), is often confused with IUGR. However, it is important to note that SGA is based on birth weight alone and does not consider in-utero fetal growth; so, infants born SGA may be constitutionally small and not have IUGR at all [2]. It is vital to differentiate between these two entities because infants with IUGR are at significantly greater risk of both short and long-term adverse outcomes. Short-term complications include hypothermia, hyperbilirubinemia, hypoglycemia, necrotizing enterocolitis (NEC), intraventricular hemorrhage (IVH), sepsis and even in utero and neonatal death [1]. In the longer term, IUGR infants are at increased risk of metabolic syndrome (obesity, type 2 diabetes mellitus, heart disease) and poor neurodevelopmental outcomes [3].

Ultrasonography (US) is the most widely used modality to assess fetal growth and it can be used to determine the fetal weight, growth velocities and dimensions, to better delineate the pattern of growth aberration [4]. This highlights the importance of neonatal and obstetric teams working together in identifying infants with true fetal growth restriction. Due to a lack of consensus in defining IUGR, it is difficult to determine the true incidence. Studies have estimated the incidence to be about 10% in all liveborn infants, though it is believed that it may be higher in stillborn infants [5].

Since abnormal development of placental vasculature resulting in poor perfusion, is the most common cause of IUGR [6]; these infants are recommended to undergo further evaluation using doppler blood flow measurements of the umbilical artery [7]. While doppler velocimetry does not predict which infants will develop IUGR; it is an indispensable tool for predicting outcomes and assessing timing of delivery in IUGR infants [8]. The validity of Doppler measurements in predicting outcomes has been extensively studied by randomized controlled trials [9-11]. Adding umbilical artery doppler velocimetry to the evaluation of infants with IUGR, decreases the rate of perinatal death by up to 29% [12]. Diminished end-diastolic flow in umbilical artery usually does not lead to poor outcomes, but absent or reversed end-diastolic velocities (AREDV) are associated with both perinatal mortality and poor neurodevelopment [13-15]. There is also a significant pathologic correlation between AREDV and poor health of the terminal villous vasculature of the placenta [16].

Extremely low birthweight (ELBW) infants (birth weight < 1,000 grams), account for only 0.7% of the total live births in the United States but have a disproportionately high contribution to neonatal morbidity and mortality [17]. However, growth restricted ELBW infants are at an even greater risk of adverse outcomes [18]. ELBW infants are also at high risk for postnatal growth failure [19] but feeding intolerance and risk of NEC are barriers to enteral feeding [20]. AREDV in the umbilical artery has been proposed to affect visceral perfusion and lead to a greater incidence of intestinal complications [21]. Thus, we compared the nutritional outcomes, postnatal growth velocities and major neonatal morbidities of ELBW IUGR infants, with and without AREDV, to appropriate-for-gestational age (AGA) controls.

## Materials and methods

We conducted a 6-year retrospective case-control study in the neonatal intensive care units (NICU) at the University of Texas Medical Branch, Galveston, and the University of Texas Health Science Center, San Antonio (January 2008 to December 2013). After institutional review board approval, we included 117 infants in our study. Our inclusion criteria were infants with birth weight < 1,000 grams and survival of at least seven days. Infants with major congenital malformations (like neural tube defects, orofacial clefts, tracheoesophageal fistulas, critical congenital heart defects, omphalocele, gastroschisis, intestinal atresia, anorectal malformations and/or renal agenesis) or chromosomal aberrations, were excluded from the study. Infants were included in the IUGR group, if the estimated fetal weight was below the 10th percentile for GA and fetal growth was less than two standard deviations below the mean, on at least two serial US. Antenatal fetal growth was estimated by measuring fetal biparietal diameter, head circumference, abdominal circumference, and femur length [22]. All IUGR infants were also evaluated with umbilical artery doppler velocimetry and the presence of AREDV on at least two serial US was considered to be a sign of placental insufficiency (PI). The control group included ELBW infants whose growth was AGA. GA was estimated by a first trimester US (8-13 weeks' gestation). Oligohydramnios was defined as an amniotic fluid index (AFI) <5th percentile for GA [23]. To eliminate confounders related to feeding protocol variability and practices between the two NICUs, each IUGR infant was matched to an AGA control, born at the same unit within one month of their date of delivery. A sample feeding protocol for low-birth-weight infants has also been included in the supplementary materials.

Maternal data including gravidity, parity, abortions, ethnicity, amniotic fluid volume, smoking during pregnancy, pregnancy-induced hypertension (PIH), and antenatal US results were collected from their electronic medical records. Neonatal data collected included GA, estimated fetal weight, sex, total enteral calorie intake at 28 days of life (DOL) and 36 weeks corrected gestational age (CGA), total duration of parenteral nutrition, and weekly weights. Neonatal morbidities recorded were NEC (stage ≥ 2), Conjugated hyperbilirubinemia (defined as direct bilirubin ≥ 2 mg/dL), Patent ductus arteriosus (PDA) (ductal size > 2.5 mm), Bronchopulmonary dysplasia (BPD) (based on the National Institute of Child Health and Human development criteria) and Retinopathy of prematurity (ROP) (stage ≥ III: severely abnormal blood vessel growth or requiring treatment). Growth velocity over the first month of life was calculated using Patel’s exponential model (Growth velocity= [1,000*ln (Wn/W1)]/Dn-D1); where 1=beginning of time interval, n=end of time interval, W=weight, and D=day) [24].

Collected data were analyzed with IBM SPSS Statistics for Windows. Mann-Whitney U test (IUGR vs AGA) and Kruskal-Wallis test (IUGR+PI vs IUGR-PI vs AGA) were used for continuous variables and Fisher’s Exact test and Pearson’s Chi-squared test were used for categorical variables. Kruskal-Wallis test was followed by Bonferroni multiple comparison testing. Results were expressed as median with interquartile range (IQR) or numbers (n) with percentage (%).

## Results

We included 117 ELBW infants in our study, of these 58 infants had IUGR, and 59 were matched AGA controls (the numbers in the two groups are different due to a set of AGA twins in the control group). IUGR infants weighed significantly less at birth compared to AGA controls (median birth weight- IUGR: 702.5 vs AGA: 820 grams, p-value 0.004), they were also older in GA (median GA IUGR: 28 vs AGA: 26 weeks, p-value < 0.001) and had a significantly higher incidence of PIH (IUGR: 66% vs AGA: 32%, p-value < 0.001), oligohydramnios (IUGR: 28% vs AGA: 10%, p-value 0.014) and conjugated hyperbilirubinemia (IUGR: 36% vs AGA: 16%, p-value 0.034). However, there were no differences between the two groups with regard to enteral calorie intake, TPN duration, or major neonatal morbidities and mortality (Table 1).

**Table 1:** Characteristics and outcomes of Intrauterine growth-restricted (IUGR) infants compared to appropriate-for-gestational age (AGA) infants. PIH - Pregnancy-induced hypertension; DOL - Days of life; CGA - Corrected Gestational age; TPN - Total parenteral nutrition; NEC - Necrotizing enterocolitis; PDA - Patent ductus arteriosus; BPD - Bronchopulmonary dysplasia; ROP - Retinopathy of prematurity; g - grams; d - days. Results are expressed as median with interquartile range or numbers (n) with percentage (%).

	IUGR (n=58)	AGA (n=59)	P-value
Gestational Age (weeks)	28 (26-29)	26 (25-27)	<0.001
Birth Weight (g)	702.5 (570-842.5)	820 (670-920)	0.004
Race, n (%)			0.6
Black	2 (3)	4(7)	
Hispanic	33 (57)	34(58)	
White	7 (12)	3(5)	
Other	16 (28)	18(30)	
Gender, n (%)			0.4
Female	29 (50)	34 (58)	
Male	29 (50)	25(42)	
Smoking, n (%)	12 (21)	6(10)	0.11
PIH, n (%)	36 (64)	19(32)	<0.001
Oligohydramnios, n (%)	16 (28)	6(10)	0.014
DOL 28 enteral calories (Kcal/Kg/day)	81 (32-116)	110 (51-129)	0.077
36 weeks CGA enteral calories (Kcal/Kg/day)	119 (83-129)	119 (109-124)	>0.9
TPN duration (d)	25 (17-38)	21 (15-34)	0.12
Conjugated hyperbilirubinemia, n (%)	20 (36)	9(16)	0.034
NEC, n (%)	8 (14)	4(7)	0.2
PDA, n (%)	19 (33)	11(19)	0.093
BPD, n (%)	28 (51)	35(59)	0.4
ROP, n (%)	9 (16)	12(20)	0.6
Death, n (%)	2 (3)	1(2)	0.6

The IUGR group was further divided based on the presence (IUGR+PI; n=23) or absence of PI (IUGR-PI; n=35). These groups were then compared to AGA controls and each other. The results of nutrition, growth, and morbidity, and mortality are as follows.

Nutrition

We found that despite being the oldest in GA (median GA IUGR+PI: 29 vs IUGR-PI: 28 vs AGA: 26 weeks, p-value <0.001), IUGR+PI infants had the lowest enteral calorie intake at DOL 28 and 36 weeks CGA compared to infants with normal placental blood flow (median intake DOL 28- IUGR+PI: 32 vs IUGR-PI: 93 vs AGA: 110 kcal/kg/day; p-value 0.011; median intake 36 weeks CGA- IUGR+PI: 102 vs IUGR-PI: 125 vs AGA: 119 kcal/kg/day; p-value 0.012). Consequently, we saw a trend towards IUGR+PI infants requiring TPN for longer durations (median duration IUGR+PI: 35 vs IUGR-PI: 25 vs AGA: 21 days; p-value 0.054) and having a higher incidence of conjugated hyperbilirubinemia (IUGR+PI: 43% vs IUGR-PI: 29% vs AGA: 16%; p value 0.058), compared to the other groups, but these results did not reach statistical significance (Table 2).

**Table 2:** Nutritional outcomes of Intrauterine growth-restricted infants with placental insufficiency (IUGR + PI) vs intrauterine growth-restricted without placental insufficiency (IUGR - PI) vs Appropriate-for-gestational age (AGA) infants. DOL - Days of life; CGA - Corrected Gestational age; TPN - Total parenteral nutrition; g - grams; d - days. Results are expressed as median with interquartile range or numbers (n) with percentage (%). * Represents p value < 0.05 IUGR + PI vs IUGR – PI. Φ Represents p value < 0.05 IUGR - PI vs AGA. † Represents p-value < 0.05 IUGR + PI vs AGA.

NUTRITIONAL OUTCOMES	IUGR+PI (n=23)	IUGR-PI (n=35)	AGA (n=59)	P-value
Gestational Age (weeks)	29 (27-30)	28 (26-29) ϕ	26 (25-27) †	<0.001
Birth Weight (g)	720 (580-804)	690 (585-845)	820 (672-920)	0.015
DOL 28 enteral calories (kcal/kg/day)	32 (8-102) *	93 (54-120)	110 (51-129) †	0.011
36 weeks CGA enteral calories (kcal/kg/day)	102 (35-120) *	125 (116-131)	119 (109-124)	0.012
TPN duration (d)	35 (18-65)	25 (16-30)	21 (15-34)	0.054
Conjugated hyperbilirubinemia, n (%)	10(43)	10(29)	9(16)	0.058

Growth

**Figure 1:**
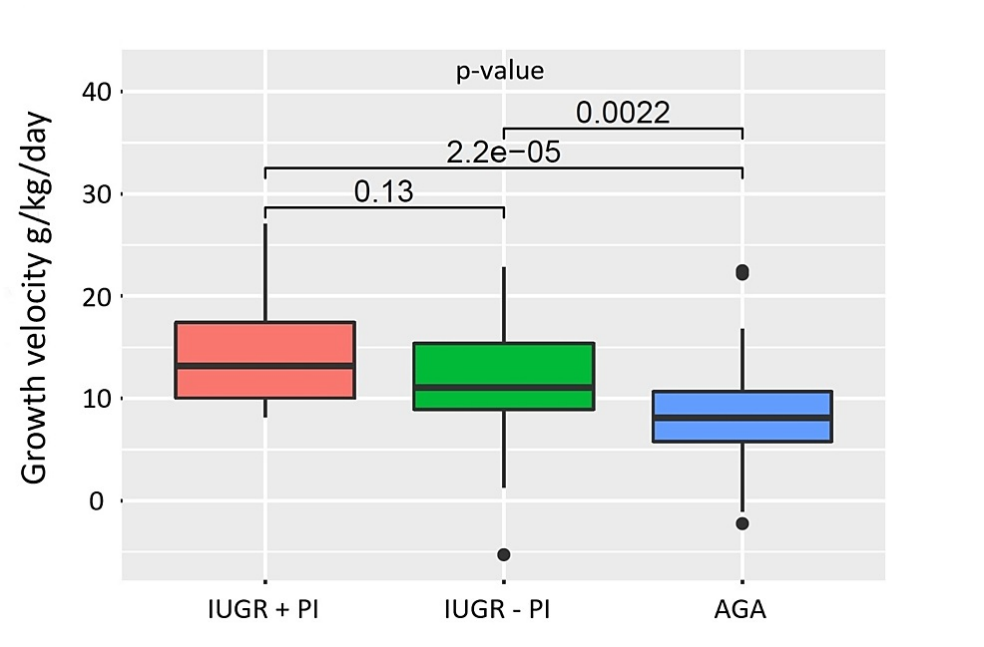
Median growth velocities of all infants over the first month of life. g - grams; kg - kilograms. Median growth velocity IUGR+PI: 13.2 (9.8-18.4) vs IUGR-PI: 11.1 (8.9-15.6) vs AGA: 8.1 (5.7-10.7) g/kg/day. Results expressed as median with interquartile range. Growth velocities were calculated using Patel’s exponential model [24].

We used Patel’s exponential model [24] to calculate and compare growth velocities over the first month of life and found that IUGR+PI infants showed good catch-up growth and surpassed the growth velocities of the other two groups over the first postnatal month (median growth velocity IUGR+PI: 13.2 vs IUGR-PI: 11.1 vs AGA: 8.1 g/kg/day, p-value <0.001) (Figure 1).

Morbidity and mortality

Finally, we compared the incidence of major neonatal morbidities and mortality between the different groups. The IUGR+PI group had the highest incidence of NEC (IUGR+PI: 22% vs IUGR-PI: 9% vs AGA: 7%, p-value 0.14) and death (IUGR+PI: 9% vs IUGR-PI: 0% vs AGA: 2%, p-value 0.2) but the lowest incidence of BPD (IUGR+PI: 33% vs IUGR-PI: 62% vs AGA: 59%; p-value 0.08) and ROP (IUGR+PI: 10% vs IUGR-PI: 20% vs AGA: 20%; p-value 0.6), but these differences did not reach statistical significance (Table 3).

**Table 3:** Major neonatal morbidities of Intrauterine growth-restricted infants with placental insufficiency (IUGR + PI) vs intrauterine growth-restricted without placental insufficiency (IUGR - PI) vs Appropriate-for-gestational age (AGA) infants. NEC- Necrotizing enterocolitis; PDA- Patent ductus arteriosus; BPD- Bronchopulmonary dysplasia; ROP- Retinopathy of prematurity. Results expressed as numbers (n) with percentage (%).

MORBIDITIES	IUGR+PI (n=23)	IUGR-PI (n=35)	AGA (n=59)	P-value
NEC, n (%)	5(22)	3(9)	4(7)	0.14
PDA, n (%)	5(22)	14(40)	11(19)	0.06
BPD, n (%)	7(33)	21(62)	35(59)	0.08
ROP, n (%)	2(10)	7(20)	12(20)	0.6
Death, n (%)	2(9)	0(0)	1(2)	0.2

## Discussion

Our study compared the outcomes of IUGR infants, with and without PI, to AGA controls in a population of ELBW infants, with a special emphasis on nutrition and growth. To our knowledge, there have been no studies in ELBW infants that have evaluated the impact of PI (as evidenced by AREDV in an umbilical artery) on nutritional outcomes.

We found that IUGR in the presence of PI was associated with lower enteral calorie intake at different clinically relevant time points, as compared to the groups with normal placental blood flow, even though these infants were the most mature in GA. To make up for the enteral calorie deficit in these infants, we also observed a trend toward the longer duration of TPN use and a higher incidence of conjugated hyperbilirubinemia, most likely secondary to parenteral nutrition-associated liver disease (Table 2).

IUGR is widely established as an adverse determinant of neonatal outcomes [1,3] and it is second only to prematurity, as a cause of neonatal morbidity and mortality around the world, making it a condition that carries a significant health burden [25]. We found that a statistically significant number of IUGR infants were born to mothers with PIH and oligohydramnios, both of which are well-established associations (Table 1) [26].

Animal studies have shown that IUGR modifies the developmental pattern of the intestines at a molecular level by changes in the cell proliferation-apoptosis balance [27]. In addition, the circulatory redistribution associated with AREDV leads to decreased blood flow to the intestines [21]. We propose that these two factors working in conjunction lead to a gut that is both structurally and functionally inferior in IUGR infants with PI and is responsible for the observation of lower enteral calorie intake in this group [21,27]. Hackett et al. compared IUGR infants with AREDV to those with the normal placental flow and found that the former were more likely to have NEC and succumb to perinatal death [28]. We observed similar trends of higher NEC, which may be one of the reasons for the lower enteral calorie intake, but due to the small number of subjects included in our study, these findings did not reach statistical significance (Table 3).

Growth is the result of a complex interplay between genetic and environmental factors and PI is the most common cause of IUGR [6]. Another interesting finding of our study was that IUGR infants with PI surpassed the growth velocities of their AGA counterparts over the first month of life. This can be attributed to the phenomena of catch-up growth. Additionally, IUGR infants with PI trended towards longer durations of TPN use in our study. Since this mode of nutrition bypasses intestinal absorption this would have contributed to their accelerated growth velocities (Figure 1).

The major weakness of our study was the retrospective nature of data collection and the relatively small sample size. Also, since data were collected across two different NICUs, it is difficult to account for the subtle variations in physician practices and nursing care between the two units. We attempted to reduce the effect of these differences on the outcomes of our study, by matching IUGR infants with AGA controls based on location and timing of delivery, which also contributed to a smaller sample size.

## Conclusions

The results of our study suggest that IUGR, in the presence of abnormal placental blood flow, is associated with significantly poorer nutritional outcomes in ELBW infants. These findings emphasize the need for further prospective studies to evaluate feeding regimens specifically tailored to meet the demands of this challenging group. This will improve the overall tolerance to enteral feeds and reduce the costs and complications associated with prolonged parenteral nutrition use and extended NICU stays.

## Figures and Tables

**Figure 2:**
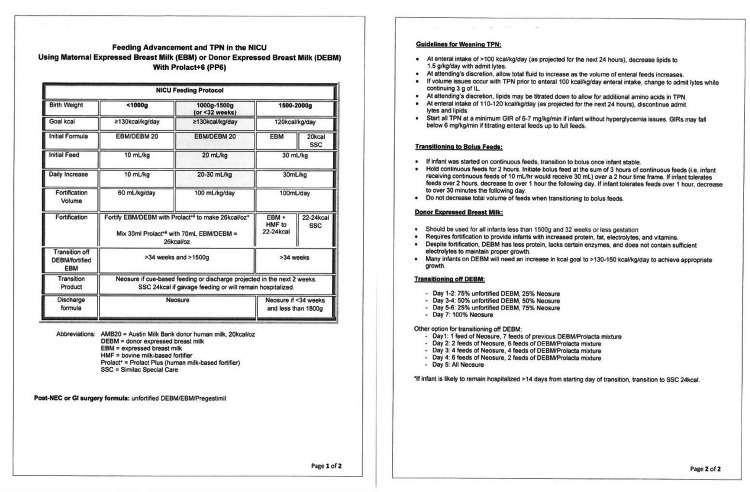
Standardized feeding protocol for low birth weight infants.
